# The Impact of Metabolic Syndrome and Obesity on the Evolution of Diastolic Dysfunction in Apparently Healthy Patients Suffering from Post-COVID-19 Syndrome

**DOI:** 10.3390/biomedicines10071519

**Published:** 2022-06-27

**Authors:** Cristina Tudoran, Mariana Tudoran, Talida Georgiana Cut, Voichita Elena Lazureanu, Felix Bende, Renata Fofiu, Alexandra Enache, Silvius Alexandru Pescariu, Dorin Novacescu

**Affiliations:** 1 Department VII, Internal Medicine II, University of Medicine and Pharmacy “Victor Babes” Timisoara, E. Murgu Square, Nr. 2, 300041 Timisoara, Romania; tudoran.cristina@umft.ro (C.T.); bende.felix@umft.ro (F.B.); 2 Center of Molecular Research in Nephrology and Vascular Disease, Faculty of Medicine, University of Medicine and Pharmacy “Victor Babeș” Timișoara, E. Murgu Square, Nr. 2, 300041 Timisoara, Romania; 3 County Emergency Hospital, L. Rebreanu Str., Nr. 156, 300723 Timisoara, Romania; renata.fofiu@umft.ro (R.F.); dorin.novacescu@yahoo.com (D.N.); 4 Academy of Romanian Scientists, Splaiul Independentei Nr. 54, 50085 Bucuresti, Romania; pescariu.alexandru@umft.ro; 5 Department XIII, Discipline of Infectious Diseases, University of Medicine and Pharmacy “Victor Babes” Timisoara, E. Murgu Square, Nr. 2, 300041 Timisoara, Romania; lazureanu.voichita@umft.ro; 6 Center for Ethics in Human Genetic Identification, University of Medicine and Pharmacy “Victor Babes” Timisoara, E. Murgu Square, Nr. 2, 300041 Timisoara, Romania; enache.alexandra@umft.ro; 7 Doctoral School, University of Medicine and Pharmacy “Victor Babes”, 300041 Timisoara, Romania; 8 Center of Advanced Research in Gastroenterology and Hepatology, Faculty of Medicine, University of Medicine and Pharmacy “Victor Babes” Timisoara, 300041 Timisoara, Romania; 9 Department VIII, Discipline of Forensic Medicine, University of Medicine and Pharmacy “Victor Babes” Timisoara, E. Murgu Square, Nr. 2, 300041 Timisoara, Romania; 10 Department VI, Cardiology, University of Medicine and Pharmacy “Victor Babes” Timisoara, E. Murgu Square, Nr. 2, 300041 Timisoara, Romania

**Keywords:** COVID-19, diastolic dysfunction, metabolic syndrome, obesity, inflammation, transthoracic echocardiography, post-COVID-19 syndrome

## Abstract

(1) Background: Coronavirus disease 2019 (COVID-19) has a worse prognosis in individuals with obesity and metabolic syndrome (MS), who often develop cardiovascular complications that last throughout recovery. (2) Methods: This study aimed to analyze the evolution of diastolic dysfunction (DD), assessed by transthoracic echocardiography (TTE), in 203 individuals with and without obesity and/or MS diagnosed with post-COVID-19 syndrome. (3) Results: DD was frequently diagnosed in patients with MS and obesity, but also in those without obesity (62.71% and 56.6%, respectively), in comparison to 21.97% of subjects without MS (*p* ˂ 0.001). Almost half of the patients with obesity and MS had more severe DD (types 2 and 3). As for evolution, the prevalence and severity of DD, particularly types 1 and 2, decreased gradually, in parallel with the improvement of symptoms, progress being more evident in subjects without MS. DD of type 3 did not show a significant reduction (*p* = 0.47), suggesting irreversible myocardial damages. Multivariate regression analysis indicated that the number of MS factors, the severity of initial pulmonary injury, and protein C levels could explain DD evolution. (4) Conclusions: DD was commonly diagnosed in individuals with post-COVID-19 syndrome, particularly in those with MS and obesity. After 6 months, DD evolution, excepting that of type 3, showed a significant improvement, mostly in patients without MS.

## 1. Introduction

As infection with the severe acute respiratory syndrome (SARS-CoV-2) virus continues to evolve into the most important pandemic of recent centuries, it has become obvious that certain individuals develop more severe forms of the disease with worse prognoses and increased mortality rates [1,2]. Observations from the early stages of COVID-19 indicated that, beside advanced age and comorbidities, risk factors for a higher mortality are metabolic diseases, such as obesity, type 2 diabetes mellitus (T2DM) and metabolic syndrome (MS) [3,4]. These dysfunctions are associated with enhanced inflammation and metabolic imbalance, which may significantly disturb the normal response of the immune system to infections, resulting in a pro-inflammatory state [3,5], and leading often to chronic inflammatory processes, explained by the well-known fact that pro-inflammatory cytokines, such as tumor necrosis factor (TNFα), interleukine-6 (IL-6), and interleukine-1β (IL-1β), are upregulated in the adipose tissue of individuals with MS. Moreover, obesity is characterized by an increased baseline expression of the IL-6 receptor, which is not downregulated by a higher level of cytokines, thus maintaining a persistent inflammatory state, called metaflammation [3]. Increased levels of cytokines inhibit insulin signaling, favoring the development of insulin resistance, which leads to an increased infiltration of adipose tissue with macrophages, especially those of type M1, a highly inflammatory subpopulation [6,7]. This mechanism explains, also, the inadequate cardiovascular risk profile of those individuals who are predisposed to develop systemic hypertension with left ventricular hypertrophy (LVH) and diastolic dysfunction (DD). In COVID-19, the exacerbated immune response is probably realized by two modalities: a direct one resulting in systemic inflammation due to an increased release of cytokines and high levels of acute phase reactants, such as C-reactive protein and ferritin, and an indirect mechanism secondary to the activation of hypoxia-inducible factor (HIF-1α) in the macrophages [8,9,10]. It has been assumed that in COVID-19 the development of DD is favored by several pathophysiological mechanisms acting during the acute phase, such as a direct effect of the virus on myocytes, inflammation, ischemia, and endothelial dysfunction, which may induce primary acute injury of the myocardium or even necrosis [11]. Throughout recovery, some of these processes persist, triggering a progressive subsequent fibrotic remodeling which leads to myocardial stiffening and alteration of cardiac relaxation [11,12]—pathophysiological hallmarks of DD—and heart failure with reduced ejection fraction (HFrEF) [11,12]. An inappropriate cardiometabolic risk profile can supplementarily contribute to the occurrence/exacerbation of DD in individuals who, prior to infection with the SARS-CoV-2 virus, would have been considered healthy [3].

During recovery from COVID-19, in some individuals, particularly those with metabolic dysfunctions, the compensatory anti-inflammatory response syndrome, which should re-establish immune homeostasis, is frequently deficient, so that immune depression and exacerbated inflammatory processes may persist, while viral reactivation could even appear, explaining the persistence of multiple symptoms designated as post-acute COVID-19 if symptoms last between 3 and 12 weeks and as long COVID-19 if symptoms persist over 3 months since the onset of the disease [11,12,13,14]. In a significant percentage of these patients several cardiovascular abnormalities can be assessed by transthoracic echocardiography (TTE) months after COVID-19 infection [15,16,17,18,19,20], explaining, at least partially, several of these symptoms [21,22].

The aim of our study was to analyze the impact of obesity and metabolic dysfunction on the development and evolution of DD in patients recovering from a SARS-CoV-2 infection, as well as their outcomes in relation to body mass index (BMI), number of factors defining MS, and physical activity status. Another aim was to follow-up the long-term evolution of post-acute and long COVID-19 syndromes.

## 2. Materials and Methods

### 2.1. Study Population

For this analitical study, we selected randomly a study group of a larger population who suffered from SARS-CoV-2 infection during the first four waves of the COVID-19 pandemic and who attended the outpatient medical services of our hospital between the first of March and the first of October 2021 for persisting symptoms, such as long-lasting fatigue, reduced exercise capacity, dyspnea, chest pain/discomfort, palpitations, increased blood pressure values, dizziness, concentration issues, foggy brain, and sleep disturbances. From among these, we selected 423 subjects, younger than 55 years, who considered themselves as apparently healthy before getting infected with the SARS-CoV-2 virus and who were hospitalized or treated as outpatients within 4 to 10 weeks previously for a mild/moderate form of this infection. After being examined, and based on their symptoms, they were all diagnosed with post-acute COVID-19 syndrome. They were proposed to undergo further medical evaluations if they agreed to participate in our study and could provide medical documents confirming infection with the SARS-CoV-2 virus, including initial chest computed tomography (CCT), electrocardiography (ECG), and blood tests. They were also asked to produce a recent medical letter (written within the last 12 months) confirming their health status and including physical exam, ECG, basal TTE, and blood test results, including fasting blood glucose level, lipid panel, and uric acid level. Only 292 individuals wanted to participate and were able to bring the required documents, and, of these, a further 54 were excluded due to underlying pathologies not declared at baseline. Two-hundred-and-thirty-eight subjects aged between 21 and 55 years remained, and they all signed an individual informed consent form prior to the collection of any data. Out of these, based on the TTE evaluation, a further 35 were excluded due to evidence of reduced LVEF and/or right ventricular dysfunction (RVD).

The inclusion criteria were as follows: subjects aged over 18 years, capable of signing informed consent, with a SARS-CoV-2 infection confirmed by a positive result for a real-time reverse transcriptase–polymerase chain reaction (RT-PCR) assay of nasal and pharyngeal swabs and a COVID-19 evaluation consisting of a CCT assessment and ECG and laboratory tests, and who self-monitored their oxygen saturation by pulse-oximetry at home. According to a previous medical assessment, they should have been apparently healthy before COVID-19, without a pre-existing history of significant cardiovascular (CV) diseases, even if they were overweight or obese and fulfilled the diagnostic criteria for MS.

Exclusion criteria: patients aged over 55 years (with a higher likelihood to suffer from chronic CV pathology or to have age-related abnormalities on the TTE), subjects who had a severe/critical form of COVID-19, those without a baseline COVID-19 evaluation, and patients with a history of pre-existing CV pathology or who were diagnosed during the study with significant heart diseases, including those with reduced LVEF and/or RVD and those without a pre-existing assessment, including ECG, TTE, and blood tests, that confirmed their health status.

The Local Scientific Research Ethics Committee of our hospital approved the design and methodology of our study (no. 206/7.09.2020).

### 2.2. Study Protocol, Clinical and Laboratory Assessments

After inclusion in the study, all data concerning the pre-COVID-19 health status of the 203 patients were recorded and analyzed based on their medical documents, with special attention being given to the record of risk factors, body weight and height, blood pressure values, and self-assessed level of physical activity (number of hours/week). Then, the results of the initial COVID-19 evaluation were collected, including CCT (describing the extent of pulmonary injury, with lesions under 30% being considered mild and those between 30 and 60% moderate), ECG, and blood tests. Subsequently, all subjects underwent a detailed clinical exam, including assessment of BMI and waist circumference, then an ECG and a comprehensive TTE were performed and blood samples for the assessment of basal blood glucose, creatinine, uric acid, total cholesterol, low-density lipoprotein (LDL) cholesterol, high-density lipoprotein (HDL) cholesterol, triglyceride, and C-reactive protein levels were obtained (CRP). According to their BMI in kg/m2, calculated based on weight and height, the subjects were classified as normal weight (18.5–24.99), overweight (25–29.99), or obese (BMI ≥ 30). Following the cardiologic and laboratory evaluations, we noticed that all obese subjects also fulfilled the diagnostic criteria for MS, and individuals with normal weight and overweight were classified as metabolically healthy and metabolically unhealthy depending on the number of criteria for MS definition met. Thus, to simplify the analysis of our study group, we divided them into three subcategories: group A: obese patients with MS, group B: normal and overweight subjects with MS, and group C: normal and overweight subjects without MS.

### 2.3. Echocardiographic Assessments

TTE was performed according to guideline recommendations [23,24,25,26], including a comprehensive evaluation of cardiac morphology and function. Afterwards, left and right ventricular performance were assessed by determining the following parameters:

(a) Left ventricular (LV) systolic performance, was evaluated in 2D mode, from the apical 2-, 3-, and 4-chamber views, by determining the LV ejection fraction (LVEF) using the modified Simpson rule (values less than 50% were considered abnormal) and the lateral mitral annular plane systolic excursion (MAPSE) measure (values under 10 mm were considered pathological). LV global longitudinal strain (LV-GLS) was quantified from apical 2-, 3-, and 4-chamber views, the region of interest being automatically generated and, after tracing the LV endocardial border, manual corrections were performed to fit the thickness of the LV myocardial wall [18,25,26]. Values under −18% suggested impaired LV systolic function (LV-SF).
(b) Right ventricular (RV) function (RVF) was determined from an apical 4-chamber view by measuring tricuspid annular plane systolic excursion (TAPSE), assessed in M-mode, at the level of the lateral tricuspid valve annulus, by calculating the fractional area change and by determining in apical 4-chamber view the RV global longitudinal strain (RV-GLS), RVD being certified by either TAPSE < 17 mm, FAC ˂ 35%, and/or RV-GLS < −28% [18,26].
(c) To appreciate the systolic pressure in the pulmonary artery (sPAP), we determined the peak tricuspid regurgitation velocity (TRV) in continuous-wave Doppler, from the apical window at the level of the tricuspid valves, and we employed Bernouli’s equation to calculate the pressure gradient, to which we added the estimated right atrial pressure, based on the inferior vena cava diameter, and its respiratory variations. We considered that sPAP values of ≥35 mmHg at rest indicated pulmonary hypertension (PH), with severities ranging from mild (35–44 mmHg) to moderate (45–60 mmHg) to severe (>60 mmHg) [23,27].
(d) To evaluate DD, the apical 4-chamber view was employed to determine the left atrial volume index (LAVI), then pulsed Doppler was used to register the peak early diastolic velocity (E), and the late diastolic velocity (A) at the level of the mitral valve annulus, and, subsequently, the E/A ratio was calculated. Afterwards, tissue Doppler imaging (TDI) was employed to record the early (e’) and the late diastolic velocity (a’) at the level of the septal and lateral mitral annulus, and an average E/e’ ratio was calculated. According to guidelines [24], DD is classified as mild or grade 1 (impaired relaxation pattern), moderate or grade 2, and severe (restrictive filling) or grade 3. An E/A ratio ≤ 0.8 and E < 50 cm/s defines DD of type 1, while an E/A ratio over 2 indicates a DD of type 3 DD. In the case of an E/A ratio ≤ 0.8 but with an E of over 50 cm/s, or an E/A ratio between 0.8 and 2, type 2 DD was presumed and certified if at least two of the following criteria were fulfilled: an average E/e’ > 14, LAVI of over 34 mL/m^2^, and/or a TRV over 2.8 m/s. If only one of these three criteria was fulfilled, a DD of type 1 was diagnosed [24].

To classify the impact of symptomatology and to assess recovery status after COVID-19, we employed the Post-COVID-19 Functional Status (PCFS) scale—a system developed to frame the severity of functional limitations. According to this method, 0 signifies “no limitations and symptoms”, 1—“negligible limitations of usual activities with persistent symptoms”, 2—“slight limitations with significant symptoms”, 3—“moderate limitations and not able to perform all usual activities due to symptoms, but still able to take care of him/herself without assistance”, and 4—“severe limitation due to symptoms and requiring assistance to take care of themselves” [28]. All study subjects were asked to self-assess their pre-COVID-19 physical activity status, meaning the number of hours per week in which they exercised (jogging, running, cycling, swimming, fitness, brisk walking).

These assessments, including the cardiologic exam and TTE only in patients diagnosed with DD and/or other cardiac abnormalities, were repeated at 3 and 6 months after the first evaluation. The Local Scientific Research Ethics Committee of our hospital approved the design and methodology of this study (no. 206/7.09.2020).

### 2.4. Statistical Methods

We employed the Statistical Package for the Social Sciences v.25 (SPSS, Chicago, IL, USA) to perform the analysis of the obtained data. We presented the continuous numerical variables which had normal distributions as means with standard deviations (SDs), while in cases of variables with non-normal distributions, median and interquartile ranges (IQRs) were used, and the categorical variables were expressed as frequencies and percentages. Due to the fact that the analysis of the normality test (Shapiro–Wilk) evidenced a non-Gaussian distribution, we performed the statistical analysis using nonparametric tests. The Mann–Whitney U test was utilized to compare our groups of patients, and Fisher’s exact test or the chi-square test were employed to appreciate the significance of differences in the proportions of nominal variables. We employed Spearman’s correlation test to evaluate the potential associations between DD, as defined by the E/e’ ratio, and the factors characterizing MS, with patient’s age, BMI, the extent of the initial lung injury as assessed on CCT, laboratory results, and several TTE parameters [29]. To identify the predictive factors that could explain the development and evolution of DD and the number of symptoms as well, we employed both univariate and multivariate regression analyses. We considered that *p*-values under 0.05 indicated statistically significant differences.

## 3. Results

This study was conducted with a group of 203 patients, 121 (59.60%) women and 82 (40.39%) men, aged between 21 and 55 years, with a mean age of 43.12 ± 8.64 years. According to their own reports and existing medical documents prior to infection with SARS-CoV-2 virus, they all were considered as apparently healthy. Depending on their BMI and the presence of MS, they were divided into three subgroups. Their clinical and laboratory data are presented in Table 1.

**Figure 1:**
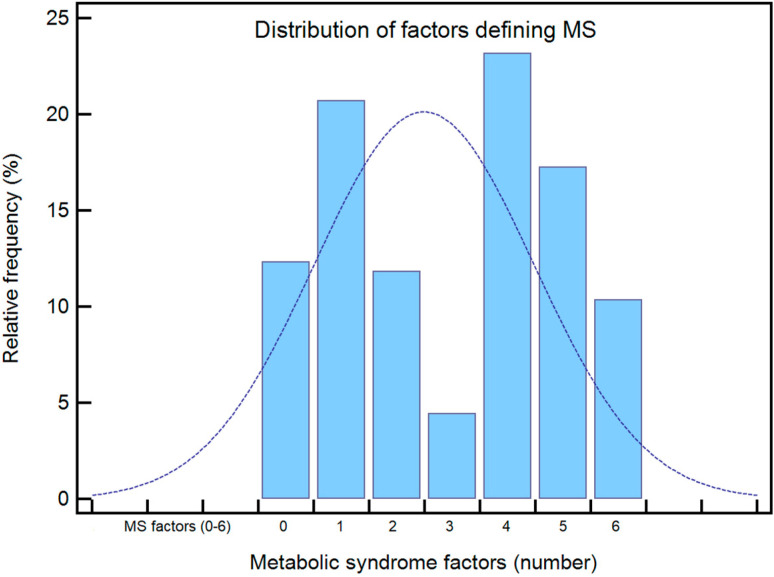
Distribution of factors defining MS.

Group A included 59 subjects, 34 (57.52%) women and 25 (42.37%) men, aged between 21 and 55 years, with a mean age of 49.55 ± 5.62 years. They all had a BMI over 30 with a median of 32.28 (30.47–33.6) Kg/m2, 51 subjects having obesity grade 1, 7 grade 2 and 1 grade 3. During acute SARS-CoV-2 infection, 41 of them (69.49%) showed evidence of lung injury, with lesions being of moderate severity in 25 patients (42.37%), a significantly higher prevalence compared to the two other groups (*p* ˂ 0.001). At the first assessment, they generally described more symptoms—a median of 6 (4–7)—and had higher PCFS levels and declared lower physical exercise levels (Table 1), all with *p* ˂ 0.001. All of the patients had MS, defined by at least three factors, but the majority of subjects had more diagnostic criteria, with a median of 5 (4–6) (Figure 1). Although within normal limits, their blood pressure values were significantly higher than those of subjects without MS (*p* ˂ 0.001).

Concerning their TTE results, although LVEF was over 50% and no significant RVD was evidenced, most subjects had various abnormalities. LVH was identified in 12 men and in 21 women. Of these, 37 (62.71%) had DD: 8 subjects with an E/A ratio < 0.8 were classified as having DD type 1, while another 4 (10.81%) had DD type 3, with an E/A ratio > 2. Among the remaining 25, with E/A ratio ≤ 0.8 but with an E > 50 cm/s or with an E/A ratio between 0.8 and 2, DD type 2 was confirmed in 14 subjects (37.83%), and the other 11 subjects, with only one criterion, were confirmed as having DD type 1, resulting in a total of 19 subjects (51.35%) with this dysfunction (Table 2), all diagnostic criteria being significantly worse than in the other categories (*p* ˂ 0.001). Pericarditis with small exudate was detected in two patients, and a thickened pericardium of over 4 mm was found in two other subjects.

Group B included 53 subjects, 33 (62.26%) women and 20 (37.73%) men, aged between 43 and 55 years, with a mean age of 46.98 ± 4.74 years, all of them with a BMI under 30 Kg/m^2^, the median value being 27.7 (26.5–29.98) Kg/m^2^. All of the patients had MS, the median number of factors being 4 (4–5) (*p* ˂ 0.001), and they had significantly higher blood pressure values than those in group C (*p* ˂ 0.001). During COVID-19 infection, 32 of them (60.37%) had pulmonary injury, but this was of moderate severity in only 12 patients, the remaining 41 subjects being diagnosed with mild forms (*p* ˂ 0.001). They were considered as having post-acute COVID-19 based on the persistence of symptoms, with a median of 5 (3–6) and a median PCFS score of 2 (1–2); they also declared lower self-assessed exercise levels (*p* ˂ 0.001).

Out of these, 13 (24.52%) subjects, 3 men and 10 women, had LVH, and 30 (56.6%) had DD: 22 subjects (73.33%) were diagnosed with DD type 1, 7 (23.33%) with DD type 2, and only 1 (3.33%) with DD type 3—these figures being significantly worse than those for group C (*p* ˂ 0.001).

Group C comprised 91 individuals, 37 men and 54 women, aged between 26 and 55 years, with a mean age of 41.67 ± 7.45 years. Their median BMI was 24.38 Kg (22.56–26.8), 61 of them having a normal BMI and 30 being overweight. Although they did not meet three criteria for the definition of MS, most of them still had one or two of the defining criteria, reduced levels of HDL cholesterol predominating (Figure 1). The majority of these patients (82.41%) had mild forms of COVID-19, they described on average 3 (3–6) symptoms, were classified as having a mean PCFS level of 1 (1–2), and declared significantly higher levels of physical activity (*p* ˂ 0.001). Concerning their TTE results, in 8 women and in 4 men LVH was determined, and 20 patients (21.97%) had DD: 12 (60%) had type 1 and 8 (40%) had type 2, with significantly better parameters and lower prevalence than in groups A and B (*p* ˂ 0.001) (Table 2).

As we analyzed the statistical significance of the associations between DD and the severity of initial infection as reflected by the extent of lung injury assessed by CCT and immune response expressed by levels of CRP and several other clinical parameters, we observed that all correlations were statistically significant (*p* ˂ 0.001); however, the correlations between the severity of COVID-19 during the acute phase, and the number of persisting symptoms, and PCFS scale levels defining the post-acute COVID-19 syndrome (see Table 3), and, surprisingly, the pre-disease exercise levels, were the most powerful, followed by the correlations with LVMI, the number of factors defining MS, and the time elapsed since the acute infection, along with age and BMI (Table 3).

Starting from the hypothesis that MS could be implicated in the pathogeny of DD encountered in the post-COVID-19 syndrome, we analyzed the possible associations between the number of factors defining MS and other clinical, TTE, and laboratory characteristics and found that the most powerful correlations were with age and BMI (as expected) but also with several echocardiographic parameters, such as LAVI, LVMI, TRV, and average E/e’ ratio, followed by PCFS scale levels and the factors characterising the severity of acute infection. All correlations were statistically significant (*p* ˂ 0.001).

**Figure 2:**
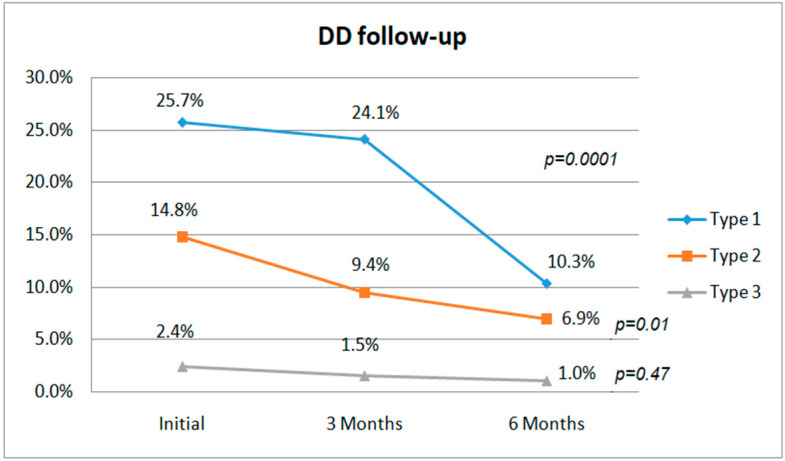
DD distribution at the initial evaluation and at 3 and 6 months after COVID-19 infection, respectively.

**Figure 3:**
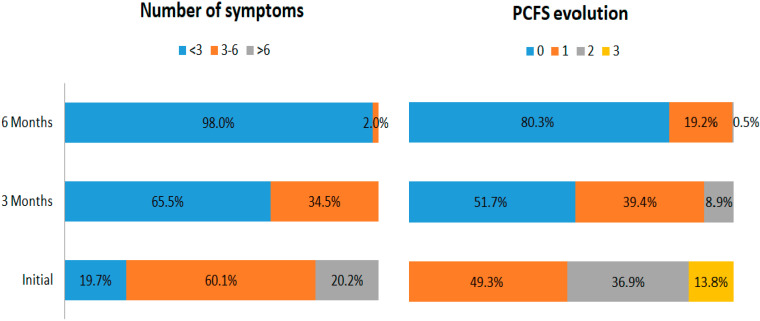
The evolution of symptom numbers and PCFS levels from the initial evaluation to 6 months after COVID-19 infection.

We followed-up the evolution of the three patient groups at 3 and 6 months and noticed an improvement in the majority of them as follows:

In group A, initially, all patients had multiple complaints, describing a median of 6 (4–7) symptoms. At 3 months, the median number of symptoms had decreased to 3 (2–3), and 9 subjects without DD assessed at the first evaluation were symptom-free (Figure 2); at 6 months, the median number of symptoms was 0 (0–2) and 34 subjects were symptom-free (Figure 3). Concerning the PCFS scale, the patients from group A were classified as having a median level of 2 (1–3); after 3 months, their status improved to a median level of 1 (1–2), with 14 subjects even being at level 0; and after 6 months, their median level was 0 (0–1), with 38 subjects at level 0 (Figure 3). Concerning DD, if initially 37 patients had DD of varying severity (16 had type 1, 17 type 2, and 4 type 3 DD), after 3 months, 34 still had DD (21 type 1, 11 type 2, and 2 type 3), and at the end of the follow-up, only 23 subjects with DD remained (13 with type 1, 8 with type 2, and 2 with type 3) (Figure 2).

In group B, although at the beginning of the study all patients had various symptoms, with a somewhat lower median of 5 (3–6) compared with group A (*p* < 0.0001), at 3 months their status improved, 13 of them being symptom-free, with a median of 2 symptoms described (0.5–3), and after 6 months, 38 individuals were asymptomatic, the median being 0 (0–1). If initially they were classified as having a median PCFS level of 2 (1–2), after 3 months, this diminished to 1 (0–1), 26 subjects achieving level 0. At the end of the follow-up, at 6 months, the median was 0 (0–1), with 44 individuals at level 1 (Figure 3). At the first TTE assessment, DD was identified in 30 subjects (22 with type 1, 7 with type 2, and 1 with type 3). After 3 months, 20 remained (14 with type 1, 5 with type 2, and 1 with type 3), and at 6 months only 9 subjects still had DD (4 with type 1 and 5 with type 2) (Figure 2).

The subjects included in group C, thus symptomatic, initially had fewer complaints, with a median of 3 (3–6) symptoms; after 3 months, this median decreased to 0 (0–2), 57 subjects being completely symptom-free; and at 6 months, 78 subjects declared the remission of symptoms, with only 13 still reporting various complaints, the median number being 0 (0–0). PCFS scale was initially 1 (1–2); at 3 months 0 (0–1), 65 patients were classified at level 0; and at 6 months, their median PCFS score was 0 (0–0), with 81 subjects being classified at level 0. DD was identified initially in 20 subjects (14 with type 1 and 8 with type 2). At the 3 months follow-up, 17 still had DD (14 with type 1 and 3 with type 2), while at 6 months, only 5 individuals with DD remained (4 with type 1 and 1 with type 2) (Figure 2).

One interesting aspect of the study concerned the 6 months’ evolution of the three types of DD: while type 1, reflecting an altered relaxation of the LV myocardium due to increased stiffening, and being characterized mostly by E/A and E/e’ ratios, decreased slightly at 3 months, a marked, statistically significant reduction (*p* ˂ 0.0001) was noticed after 6 months, suggesting an improvement in LV filling. Concerning type 2, we observed a significant reduction both at 3 and 6 months (*p* = 0.01), probably reflecting reduction in TRV for the most part but also an enhancement of LV compliance. On the contrary, for type 3 DD, no statistically significant evolution was observed, sustaining the hypothesis that there could be irreversible myocardial changes accompanied by elevated filling pressures, increased LAVI, and/or TRV. This alteration prevailed in group A, with a BMI > 30 Kg/m^2^ and MS (10.81% of patients with DD), explaining the worse evolution of DD and therefore the symptoms, also, in this group, in contrast to group B, in which DD type 3 was diagnosed in only one subject (3.33%), with none being diagnosed in group C.

In the univariate regression analysis, the following parameters were associated with the numbers of persisting symptoms defining both post-acute COVID-19 syndrome and the presence of DD, along with average E/e’ values: age (*p* < 0.001), BMI (*p* < 0.001), the number of MS factors (*p* < 0.001), CRP levels (*p* < 0.001), pulmonary injury on CCT (*p* < 0.001), weeks since COVID-19 infection (*p* < 0.001), PCFS (*p* < 0.001), SBP values (*p* < 0,0001), DBP values (*p* < 0.0001), and LVMI (*p* < 0.0001).

We employed the multivariate regression analysis to identify the independent factors that could predict the development of DD, as well as the factors influencing E/e’ values, both at the initial evaluation and at 6 months after COVID-19 infection. The regression model was built based on the forward stepwise method, and, to optimize the model, factors already known to be associated with DD (age, gender, and BMI) were excluded from the beginning (Table 4). After excluding age, gender, and BMI, the model that included the number of MS factors (*p* = 0.0004, β = 0.080 ± 0.022), pulmonary injury on CCT (*p* < 0.0001, β = 0.035 ± 0.0028), SBP values (*p* = 0.0119, β = 0.007926 ± 0.003122), and LVMI values (*p* = 0.0107, β = 0.00606 ± 0.002353) was associated with the presence of DD. E/e’ values were associated via multivariate regression analysis with the model including the following factors: LVMI values (*p* = 0.0001, β = 0.0236 ± 0.0059), CRP levels (*p* < 0.0001, β = 0.1139 ± 0.01166), and PCFS (*p* = 0.0002, β = 0.6048 ± 0.157) (Table 5).

## 4. Discussion

Among the multi-systemic complications observed throughout the evolution of COVID-19, a wide spectrum of cardiovascular manifestations have frequently been diagnosed, during both acute infection and recovery [15,30,31,32]. Apart from the highly suggestive presentations characterizing acute heart failure, acute myocardial ischemia, pulmonary thromboembolism, or several arrhythmias, the manifestations suggesting DD are more discreet, which is why its diagnosis is frequently overlooked and why it is less debated in the medical literature [18,33]. Although its symptomatology is commonly associated with heart failure with preserved ejection fraction (HFpEF), it may be confounded with the respiratory manifestations presented during infection with the SARS-CoV-2 virus. The TTE diagnosis of DD is sometimes more laborious, requiring more assessments according to guidelines [24], with some of the findings characterizing DD, such as elevated TRV, being potentially encountered in respiratory diseases associated with pulmonary hypertension and in the lung injuries common in cases of COVID-19 [33]. DD and COVID-19 share common pathophysiological pathways and have similar cardio-metabolic risk profiles. Initial remarks from the United States evidenced that people with obesity (a BMI over 30 Kg/m^2^) have a higher risk of developing more severe complications due to infection with the SARS-CoV-2 virus and are two times more likely to be admitted to an ICU compared to individuals with normal BMI (20–25)—an observation confirmed by several studies [8,33,34,35]. This risk was found to increase in parallel with the progression of obesity and was associated with higher mortality and long-term sequelae, favoring the occurrence of sub-acute and long COVID-19 syndromes even in younger patients [32,35,36,37]. This aspect is particularly relevant considering the high age-adjusted prevalence of obesity in Western countries, estimated at 42.4% in the United States, 28% in the United Kingdom, and even higher in Eastern European countries, compared with lower rates in Asian nations [11]. Moreover, it has been observed that even in younger individuals, considered as apparently healthy, the presence of MS, whether or not associated with obesity, represents a supplementary risk factor for the development of cardiovascular complications, which often persist during recovery from COVID-19 and explain, at least partially, the residual symptomatology [9,36,38,39].

The presence of DD, assessed by TTE, in patients without previous cardiovascular pathology recovering from COVID-19 who have not been diagnosed with altered LVF during the acute illness is a complex issue, with reports of the prevalence of DD varying widely, from around 16%—the figure first reported by Szekely [18]—to higher values (39%) in other studies [33], though if we consider data from magnetic resonance imaging (MRI) studies, even higher prevalences (60–78%) have been reported [40]. It is well known that obesity is also associated with an increased risk of developing DD, the prevalence of which is around 19%; among overweight subjects, the prevalence is approximately 12% compared with only 2% among normal-weight individuals [41]. MS further increases the risk of developing DD [42,43]. For this reason, we studied in our research the characteristics and evolution of DD, assessed by TTE, in 203 patients suffering from post-acute COVID-19 syndrome who were considered as apparently healthy prior to infection. They all were aged under 55 years, to avoid the impact of age on the occurrence of DD, and were grouped according to the presence of MS and BMI into three subcategories. More women than men suffered from post-acute COVID-19. The subjects with obesity and MS were somewhat older and had higher blood pressure values (though still within normal limits) than those with a BMI under 30 and MS, though their values were even higher than those of patients without MS (*p* < 0.0001), who also reported fewer symptoms, had lower PCFS levels, and suffered from less severe COVID-19 forms. Referring to the results of the TTE assessment, the patients with obesity and MS had significantly higher LVMI values (*p* < 0.0001), and all parameters defining DD (LAVI, TRV, and especially E/e’ ratio) were considerably more elevated (*p* < 0.0001) than in subjects with a BMI under 30 Kg/m2 and MS, and this difference was even more evident when considering the individuals without MS. Thus, DD prevalence in obese and even non-obese patients with associated MS and suffering from post-COVID-19 syndrome was more than double (62.71%, respectively 56.6%) what it was in those without MS (21.97%). This difference was even higher when compared to data in the literature concerning the prevalence of DD in obese subjects aged under 60 years prior to the outbreak of the COVID-19 pandemic [41] or in individuals without post-COVID-19 syndrome [44].

Referring to the evolution of DD in patients with post-acute and long COVID-19 syndrome, it is assumed that it becomes less severe as the amount of time elapsed since acute infection increases. There have been several studies on this topic [31,41,45,46], and others are under development or being planned [47]. The number and intensity of symptoms defining the post-acute and long COVID-19 syndromes, as well as levels of the PCFS scale, reduced during the 6 months of follow-up in parallel with improvement in DD. An interesting aspect, evidenced in our study, was that the prevalence of types 1 and 2 DD diminished gradually and statistically significantly (*p* < 0.0001) during the 6 months of follow-up, based especially on reductions in TRV and E/e’ ratios, while for type 3 the reduction was small and not significant from a statistical point of view (*p* = 0.47). This evolution could be explained by the fact that types 1 and 2 reflect milder, potentially reversible forms of DD, while type 3 suggests more advanced, irreversible changes or perhaps the worsening of an underlying, undiagnosed DD prior to the pandemic.

It is worth mentioning that in our study the results of the univariate regression analysis indicated that the parameters associated both with the number of persisting symptoms defining post-acute COVID-19 syndrome and the presence of DD were BMI, number of MS factors, initial CRP levels, and extent of lung injury assessed on CCT, the number of weeks elapsed since COVID-19 infection, levels of PCFS, blood pressure values, and LVMI (*p* < 0.0001). In multivariate regression analysis, after the exclusion of age and BMI, average E/e’ values were significantly influenced by LVMI values (*p* = 0.0001), CRP levels (*p* < 0.0001), and PCFS levels (*p* = 0.0002). These results evidence the impact of MS and BMI on the evolution of symptomatology and DD in patients suffering from post-COVID-19 syndrome.

The heterogeneous diagnostic criteria of DD are responsible for the main limitation of our study because we did not have a rigorous TTE assessment of all parameters required for the evaluation of DD in all patients before they were infected with the SARS-CoV-2 virus. We included only subjects with a previous TTE assessment, but, in most cases, it was an abbreviated one, mentioning only “within normal limits”, mild LVH, and eventually the E/A ratio, but without assessments of E/e’ ratio, LAVI, TRV, and LVMI. Therefore, we cannot be certain whether in our patient groups some subtle alterations, such as LVH, increased LAVI, or even DD, preceded COVID-19, and eventually worsened during this disease. Another limitation is that TRV, an important element in the diagnosis of DD, could have increased as a consequence of lung injury, taking into account that many of our patients, especially those with obesity and MS, had pulmonary involvement.

## 5. Conclusions

Our study attempted to evidence the common association between diastolic dysfunction and infection with the SARS-CoV-2 virus. This dysfunction can be detected by echocardiography more frequently than expected, even in individuals suffering from post-acute and long COVID-19 syndromes, particularly among those with MS and obesity. In most cases, the evolution of DD is favorable; its echocardiographic parameters evidence significant improvements in parallel with reductions in the number and severity of persisting symptoms, especially in individuals without MS and obesity. In this category of patients, we diagnosed severe forms of DD (type 3) more frequently, and they had worse evolutions, with advances made being minimal, suggesting irreversible cardiac damages, such as interstitial fibrosis. This observation suggests that people with increased BMI and MS should undergo a more comprehensive evaluation, including TTE, in order to diagnose DD at an earlier stage, when it could be reversible.

## Figures and Tables

**Table 1:** Clinical and laboratory characteristics of the study group.

	Group A N = 59	Group B N = 53	Group C N = 91	*p*-Value		
				A/B	B/C	A/C
Gender:						
Men	25 (42.37%)	20 (37.73%)	37 (40.65%)	0.7388	0.3858	0.7288
Women	34 (57.62%)	33 (62.26%)	54 (59.34%)	0.7588	0.9114	0.9954
Age (year)	49.55 ± 5.62	46.98 ± 4.74	41.67 ± 7.45	0.0106	<0.0001	<0.0001
BMI (Kg/m^2^)	32.28 (30.47–33.6)	27.7 (26.5–29.98)	24.38 (22.56–26.8)	<0.0001	<0.0001	<0.0001
Waist circumference (cm)	100 (94–110)	90 (89–103)	86 (78–98)	<0.0001	0.0002	<0.0001
SPB (mmHg)	130 (130–140)	130 (120–132.5)	120 (100–120)	0.0234	<0.0001	<0.0001
DBP (mmHg)	80 (70–90)	80 (70–80)	70 (60–70)	0.0133	<0.0001	<0.0001
Heart rate (b/min)	75 (70–80)	75 (75–80)	80 (75–85)	0.5030	0.0017	0.0004
Weeks since COVID-19 infection	8 (8–10)	8 (8–10)	9 (9–10)	0.8324	0.0147	0.0329
Number of symptoms	6 (4–7)	5 (3–6)	3 (3–6)	0.0962	0.0162	<0.0001
PCFS scale	2 (1–3)	2 (1–2)	1 (1–2)	<0.0001	0.0044	<0.0001
Self-assessed exercise level	0.5 (0.4–1)	1.1 (1–2)	2.3 (1.5–3)	<0.0001	<0.0001	<0.0001
**Results of the initial COVID-19 evaluation**						
COVID-19 severity:						
Moderate	25 (42.37%)	12 (22.64%)	16 (17.58%)	0.0439	0.6019	0.0017
Mild	34 (57.62%)	41 (77.35%)	75 (82.41%)	0.0439	0.6287	0.0030
Lung injury on CCT (%)	15 (15–30)	8 (0–29)	0 (0–6)	0.1737	0.0023	<0.0001
CRP (mg/dL)	30.28(27.6–36.75)	27.89 (23.9–36.4)	26.23(16.84–30.2)	0.0686	0.0119	<0.0001
**Laboratory results at baseline**						
Basal blood glucose (mg/dL)	104 (100 -118)	100 (100–110)	90 (89–95)	0.0026	<0.0001	<0.0001
Uric acid (mg/dL)	7.6 (7.3–8)	7.3 (7.2–7.5)	6.3 (6–6.8)	0.0019	<0.0001	<0.0001
LDL cholesterol (mg/dL)	140 (130–150)	120 (120–140)	100 (90–120)	<0.0001	<0.0001	<0.0001
HDL cholesterol (mg/dL)	30 (30–35)	35 (30–40)	45 (40–50)	0.0917	<0.0001	<0.0001
Triglycerides (mg/dL)	170 (160–190)	160 (160–170)	140 (130–145)	<0.0001	<0.0001	<0.0001

Legend: Group A—59 subjects with BMI ≥ 30 Kg/m^2^ and MS; Group B—53 patients with BMI ˂ 30 Kg/m^2^ and MS; Group C—91 subjects with BMI ˂ 30 Kg/m^2^ without MS; BMI—body mass index; SBP—systolic blood pressure; DBP—diastolic blood pressure; PCFS—Post-COVID-19 Functional Status scale; CCT—chest computed tomography; CRP—C reactive protein; LDL—low-density lipoprotein; HDL high-density lipoprotein.

**Table 2:** Results of the initial TTE assessment.

	Group A N = 59	Group B N = 53	Group C N = 91	*p*-Value		
				A/B	B/C	A/C
LVMI (g/m^2^)	100 (95.98–114.53)	96.12(88.3–108)	87.7 (70.45–97.54)	0.0011	0.0002	<0.0001
LVEF (%)	53 (50–55)	55 (52.5–60)	60 (55–65)	0.0025	0.0002	<0.0001
MAPSE lateral (mm)	14 (12–16)	15 (12–16)	17 (15–18)	0.5595	<0.0001	<0.0001
LV-GLS (%)	−19 (−20–−18)	−20 (−21–−19)	−21 (−22–−19)	0.0002	0.2227	<0.0001
LAVI (mL/m^2^)	30.21(23.43–30.67)	20 (17.6–27.23)	15.76 (13.3–21.34)	<0.0001	0.0002	<0.0001
E/A	0.98 (0.81–1.29)	1.01 (0.77–1.27)	1.11 (0.9–1.34)	0.8429	0.1397	0.2263
E/e’ average	14.12 (12.23–14.4)	12.83 (11.4–14.14)	11.94 (9.8–13)	0.0098	0.0023	<0.0001
TRV max (m/s)	2.69 (2.6–2.87)	2.67 (2.41–2.71)	2.51 (2–2.7)	0.0400	0.0045	<0.0001
sPAP (mmHg)	33.94 (32–37.94)	33.5 (28.23–34.37)	30.2 (21–34.16)	0.0400	0.0045	<0.0001
TAPSE lateral (mm)	20 (17–21)	20 (19–22)	24 (21–26)	0.0079	<0.0001	<0.0001
FAC (%)	36.56 (35.47–37.9)	36.23 (35–37.89)	37.89 (35.7–39)	0.8841	0.0042	0.0002
RV-GLS (%)	−28 (−30–−27)	−29 (−30–−28)	−31 (−33–−29)	0.5364	<0.0001	<0.0001

Legend: Group A—59 subjects with BMI ≥ 30 Kg/m^2^ and MS; Group B—53 patients with BMI ˂ 30 Kg/m2 and MS; Group C—91 subjects with BMI ˂ 30 Kg/m2 without MS; LVMI—left ventricular mass index; LVEF—left ventricular ejection fraction; MAPSE—mitral annular plane systolic excursion; LV-GLS—left ventricular global longitudinal strain; LAVI—left atrial volume index; E/A—peak mitral inflow early (E) to late (A) diastolic velocities ratio in pulsed Doppler; E/e′—early mitral inflow diastolic velocity E to average e′ velocity (E/e′) in pulsed tissue Doppler ratio; TRV—peak tricuspid regurgitation velocity; sPAP—estimated systolic pressure in the pulmonary artery; TAPSE—tricuspid annular plane systolic excursion; FAC—fractional area change; RV-GLS—right ventricular global longitudinal strain.

**Table 3:** Correlations between average E/e’ ratio and several clinical and laboratory data for the study patients.

	Age	BMI	MS Factors	CCT Injury	CRP	Weeks	Number of Symptoms	PCFS Scale	LVMI	Level of Exercise
R	0.46	0.36	0.46	0.63	0.75	−0.49	0.61	0.63	0.52	−0.63
95%CI	0.347–0.564	0.237–0.477	0.345–0.563	0.542–0.708	0.687–0.807	−0.589–−0.380	0.524–0.696	0.548–0.712	0.413–0.615	−0.708–−0.541
*p*	˂0.001	˂0.001	˂0.001	˂0.001	˂0.001	˂0.001	˂0.001	˂0.001	˂0.001	˂0.001

Legend: BMI—body mass index; MS—metabolic syndrome; CCT—chest computed tomography; CRP—C reactive protein; PCFS—Post-COVID-19 Functional Status scale; LVMI—left ventricular mass index; R—correlation index; CI—confidence interval; *p*—statistical significance; Spearman’s correlation.

**Table 4:** Correlations between the number of MS elements with clinical and other TTE parameters and laboratory data for the study patients.

	Age	BMI	CCT Injury	CRP	Weeks	Number of Symptoms	PCFS Scale	LVMI	E/e’ Average	TRV	LAVI
R	0.695	0.684	0.414	0.446	−0.359	0.280	0.519	0.554	0.461	0.518	0.580
95%CI	0.616–0.760	0.603–0.751	0.293–0.522	0.329–0.550	−0.474–−0.233	0.149–0.403	0.410–0.612	0.450–0.642	0.345–0.563	0.410–0.512	0.480–0.664
*p*	˂0.001	˂0.001	˂0.001	˂0.001	˂0.001	˂0.001	˂0.001	˂0.001	˂0.001	˂0.001	˂0.001

Legend: BMI—body mass index; CCT—chest computed tomography; CRP—C reactive protein; PCFS—Post-COVID-19 Functional Status scale; LVMI—left ventricular mass index; E/eꞌ—early mitral inflow diastolic velocity E to average e′ velocity (E/e′) in pulsed tissue Doppler ratio; TRV—peak tricuspid regurgitation velocity; LAVI—left atrial volume index; R—correlation index; CI—confidence interval; *p*—statistical significance; Spearman’s correlation.

**Table 5:** Multivariate linear regression analysis.

Variable	β	±SE	*p*
**Multivariate linear regression analysis of DD**			
Pulmonary injury on CCT	0.035	±0.0028	<0.0001
SBP values	0.007926	±0.003122	0.0119
MS factors	0.080	±0.022	0.0004
LVMI values	β = 0.00606	±0.002353	*p* = 0.0107
**Multivariate linear regression analysis of E/e’ values**			
LVMI values	0.0236	±0.0059	0.0001
CRP levels	0.1139	±0.01166	<0.0001
PCFS	0.6048	±0.157	0.0002

CI—confidence interval; β—regression coefficient; SE—standard error; *p*—statistical significance; TCT—thorax computer tomography; SBP—systolic blood pressure; MS—metabolic syndrome; LVMI—left ventricular mass index; CRP—C reactive protein; PCFS—Post-COVID-19 Functional Status scale.

## Data Availability

Our data are available on Mendeley, at https://doi.org/10.17632/v5nvysr84k.1.
